# Knee position at the moment of bone bruise could reflect the late phase of non-contact anterior cruciate ligament injury rather than the mechanisms leading to ligament failure

**DOI:** 10.1007/s00167-021-06470-6

**Published:** 2021-03-03

**Authors:** Alberto Grassi, Piero Agostinone, Stefano Di Paolo, Gian Andrea Lucidi, Luca Macchiarola, Marco Bontempi, Gregorio Marchiori, Laura Bragonzoni, Stefano Zaffagnini

**Affiliations:** 1 Clinica Ortopedica e Traumatologica II, IRCCS Istituto Ortopedico Rizzoli, Bologna, Italy; 2 Dipartimento di Scienze per la Qualità Della Vita QUVI, Università di Bologna, Bologna, Italy; 3 Laboratorio di Scienze e Tecniche Chirurgiche, IRCCS Istituto Ortopedico Rizzoli, Bologna, Italy; 4 Dipartimento di Scienze Biomediche e Neuromotorie DIBINEM, Università di Bologna, Bologna, Italy

**Keywords:** ACL injury, Bone bruise, Bone edema, Injury mechanism, Biomechanics, In-vivo kinematics, RSA

## Abstract

**Purpose:**

The aim of the present study was to trace knee position at the time of bone bruise (BB) and investigate how much this position departed from the knee biomechanics of an in vivo flexion–extension.

**Methods:**

From an original cohort of 62 patients, seven (11%) presented bicompartmental edemas and were included in the study. 3D models of bones and BB were obtained from MRI. Matching bone edemas, a reconstruction of the knee at the moment of BB was obtained. For the same patients, knee kinematics of a squat was calculated using dynamic Roentgen sterephotogrammetric analysis (RSA). Data describing knee position at the moment of BB were compared to kinematics of the same knee extrapolated from RSA system.

**Results:**

Knee positions at the moment of BB was significantly different from the kinematics of the squat. In particular, all the patients’ positions were out of squat range for both anterior and proximal tibial translation, varus–valgus rotation (five in valgus and two in varus), tibial internal–external rotation (all but one, five externally and one internally). A direct comparison at same flexion angle between knee at the moment of BB (average 46.1° ± 3.8°) and knee during squat confirmed that tibia in the former was significantly more anterior (*p* < 0.0001), more externally rotated (6.1 ± 3.7°, *p* = 0.04), and valgus (4.1 ± 2.4°, *p* = 0.03).

**Conclusion:**

Knee position at the moment of Bone bruise position was out of physiological in-vivo knee range of motion and could reflect a locked anterior subluxation occurring in the late phase of ACL injury rather than the mechanism leading to ligament failure.

**Level of evidence:**

Level IV

## Introduction

Mechanism of noncontact anterior cruciate ligament (ACL) injuries represents an enormously debated topic in Sports Medicine [2, 4, 10, 13, 18–20, 22, 24, 26]. Several multidisciplinary technologies have been utilized to support the many theories proposed [2, 10, 13, 20, 22]. Among them, the patterns of bone bruises (BB) that are typically found in MRI after ACL injuries have been interpreted as “hints” or “footprints” of a tibiofemoral contusion occurring during the ACL rupture mechanism [24, 30]. Although their assessment has been considered providing valuable insight into knee position near the time of ACL rupture [5, 17, 21, 23, 24, 30], whether the impact occurs during or after ACL rupture is still unknown.

Two previous studies [17, 25] investigated tibiofemoral position leading to ACL tear through tridimensional models based on the Magnetic Resonance of ACL injured knees superimposing femoral and tibial BB. Despite the novel design, both studies compared the predicted ACL rupture position with the unloaded non-weightbearing position during MRI. Comparing the tibiofemoral position near ACL injury to the weightbearing status or even during dynamic tasks would represent a substantial improvement in the understanding of ACL injury dynamics based on the study of BB [14, 15].

Therefore, the present study aimed to identify the tibiofemoral position at the time of BB in patients who occurred noncontact ACL tear and investigate how much this knee position departed from the kinematics of a physiological in vivo flexion–extension. On the bases of previous studies on BB 3D modeling [17, 25], it was hypothesized that the position and orientation of the knee at the moment of BB involve knee extension, anterior tibial translation, internal rotation and valgus, and that those values are not comprised within the ranges of motion occurred during a weightbearing flexion–extension.

## Materials and methods

This study obtained the approval from Institutional Review Board (IRB) of Rizzoli Orthopaedic Institute (ID: 40/CE/US/ml Clinical Trial Gov ID: NCT02323386). All subjects signed informed consent before participating in the study.

This study represents the secondary analysis of data collected from a prospective study aimed to evaluate the outcome of ACL reconstruction. Based on the study protocol of this prospective study, 62 patients were included and assessed preoperatively with 1.5T MRI analysis and dynamic RSA. The inclusion criteria for the original study were:

- Age 16–50 years.
- Complete, traumatic, and unilateral ACL injury.
- No previous knee ligament reconstruction or repair.
- No concomitant lesions of other ligaments.
- Absence of mild or advanced knee osteoarthritis (Kellgren–Lawrence III–IV).

For the purpose of the present study, only the patients fulfilling the following criteria were selected and further analyzed:

- Noncontact ACL injury.
- MRI-to-injury time < 4 months.
- Tibial and femoral BB on both medial and lateral compartment.
- Complete evaluation with dynamic RSA.

Of the 62 patients included in the original study, 35 had an MRI performed within 4 months from injury, and only 7 (11%) presented both medial and lateral tibiofemoral BB. All those patients experienced a noncontact ACL injury. The patients’ age ranged from 16 to 30 years, and the injury-to-MRI time ranged from 0.7 to 3.9 months. Three patients had medial meniscus lesions; one had both medial and lateral menisci lesions (Table 1).

**Table 1:** Demographic data, mean ± SD [range]

Age (years)	19 ± 5 [16–30]
Gender (M/F)	6/1
Injured leg (R/L)	4/3
Injury-to-MRI (months)	2.2 ± 1.1 [0.7–3.9]
Meniscal lesion (Y/N)	4/3
BMI	22.2 ± 2.7 [19.7–26.9]

*M* male, *F* female, *R* right, *L* left, *Y* yes, *N* no

The design of the study was aimed to identify the tibial and femoral position at the time of BB and to compare it to the joint kinematics during an active squat. For this purpose, MRI scans were used to create 3D models of the distal femur, proximal tibia bones, and BB. Subsequently, the bone models were matched according to the BB position. The BB on both compartments were necessary to achieve the best matching possible: model position based only on one single compartment edema would have neglected the rotatory parameters, especially internal–external and varus–valgus. Matching the BB, the knee position at the instant the joint extremities were supposed to come into contact and generate edema was reproduced, according to a methodology validated in a previous study [17]. The same bone models were used to calculate the knee kinematics of a squat performed on the injured limb through a dynamic RSA setup. Therefore, the knee position at the moment of BB was compared to the kinematical data of the squat.

### Bone modeling and positioning

**Fig. 1:**
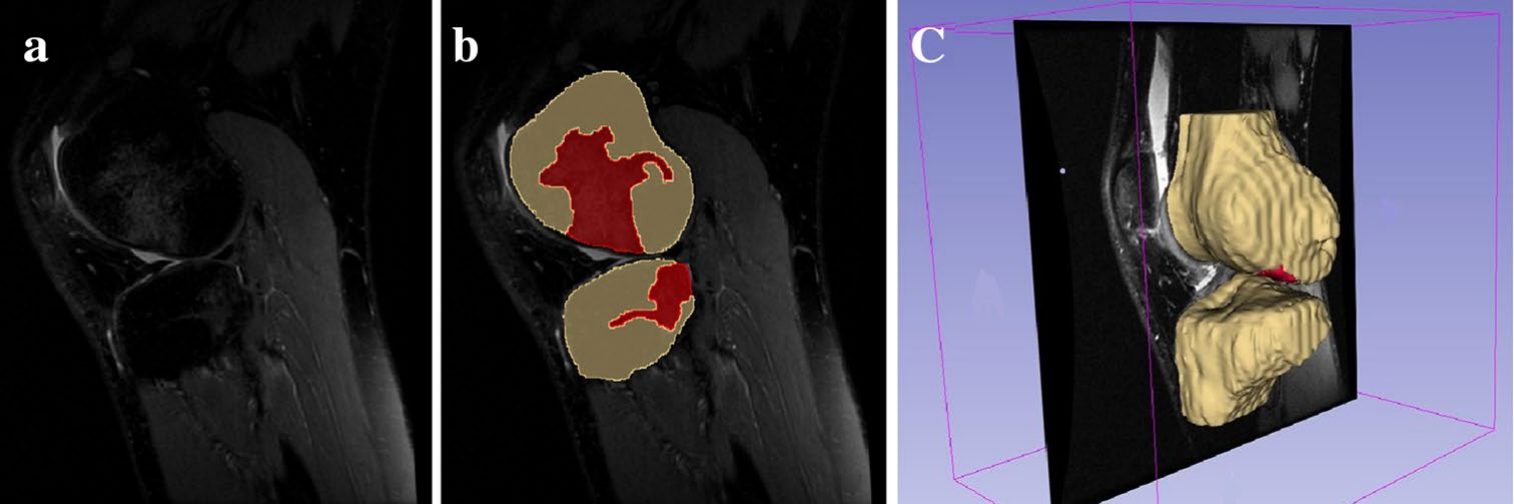
Example of segmentation process in 3D Slicer software [11]. BB were identified on MRI image of the knee (**a**) and underlined in every slice through segmentation tool (**b**), at the end of the process three-dimensional models of tibia, femur and correspondent areas with edema were obtained (**c**)

Two investigators identified the MRI matching of the inclusion criteria (P.A. and G.A.L.). The segmentation of bone surfaces and BB were performed using dedicated software (Slicer 4.10.1, Slicer, Brigham and Women's Hospital, Harvard University, NIH) [11]. Thus, 3D models of the bones (distal femur and proximal tibia) and their corresponding BB were created for each patient. The sequence used to create the models was PD fat sat, which allowed a better evaluation of the bone marrow edema. A single experienced investigator performed all the segmentations (P.A.) (Fig. 1). Subsequently, an orthopedic surgeon (A.G.) and a biomedical engineer specialized in knee pathologies (S.D.P.) reviewed the entire segmentation process.

**Fig. 2:**
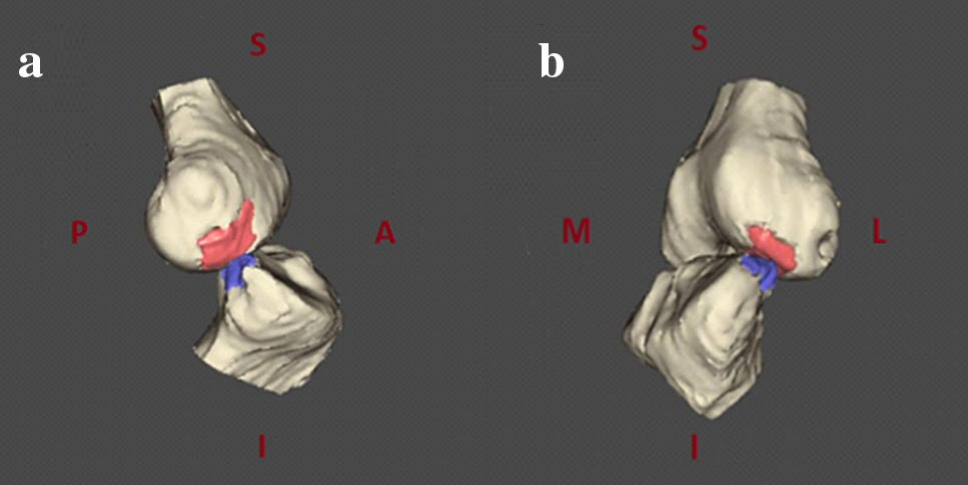
Example of 3D models positioning matching BB areas in nmsBuilder [27]: lateral view (**a**) and 45° frontal view (**b**)

The 3D models were imported in software designed to model three-dimensional geometries and subject-specific reference systems implementation (nmsBuilder v2.0, Rizzoli Orthopaedic Institute, Bologna) [27]. This software allows creating an anatomical reference system on tibia and femur 3D models according to ISB recommendation [28]. The 3D position of the tibia and femur at the moment BB was reconstructed in nmsBuilder: through the “Transform” operation command, BB was matched based on the maximal possible congruency between their external surfaces (Fig. 2). Three different investigators (P.A., A.G., and S.D.P.) reviewed the results. Finally, the software output describing the position and orientation of the tibia referred to the femur at BB was obtained.

### Dynamic RSA

The kinematical data of the squat were collected using a biplane radiographic setup for dynamic RSA. The specifics of the RSA radiographic setup were analogous to the ones already published in previous articles [1, 3, 8, 9]. The radiographic images were processed in a dedicated software in Matlab^®^ (R2016a, MathWorks Inc., Natik, MA, USA) for dynamic RSA. A 3D virtual environment was used for semi-automatic segmentation of bone contours on radiograph images and, subsequently, to place the bone models obtained from MRI according to the contours. The measurement accuracy of the validated dynamic RSA software is sub-millimetric (0.22 ± 0.46 mm and 0.26° ± 0.2° for the model position and orientation respectively, according to the ISO-5725 regulation [ISO]), as evaluated in previous studies [1, 7]. The operator’s repeatability (test–retest reliability) was evaluated through repeated tests under different image noise conditions [7]. The average error [6] was lower than 0.48 mm (95% CI 0.15–0.80 mm) for all the conditions.

### Comparison between bone bruises position and squat kinematics

The position obtained for the BB models were compared to the kinematical data obtained from RSA for the squat for each patient. Since the data were referred to the same coordinate system based on the anatomical landmarks, it was possible to compare all the knee joint kinematical parameters directly: flexion angle, internal–external (IE) rotation, varus–valgus (VV) rotation, antero-posterior (AP) translation, and proximo-distal (PD) translation.

### Statistical analysis

Knee position at the moment of BB was compared with the entire range of motion of the single-leg squat for all the kinematics parameters, separately for each patient. This way, it was possible to assess how the knee position at the moment of BB departed from the range of motion of the squat in ACL-deficient condition. Furthermore, a matched-pair comparison was performed based on the flexion angle of the BB models. The frame of the squat with the same flexion angle as the BB models was isolated. All the kinematical parameters of the squat at that frame were compared to their corresponding on the BB models, using the paired t-test. Differences were considered statistically significant for *p *< 0.05. An a-priori power analysis was performed based on G*Power (v3.1, Brunsbüttel, Germany) on the results of a previous similar study [17]. Considering a standard deviation of 14 mm and a mean difference of 22 mm between knee position at the time of BB and MRI in terms of AP translation [17], at least 7 subjects were required to have a power of 0.9 and a type I error of 0.05.

Furthermore, a post-hoc power analysis was performed to ensure the statistical effectiveness of the differences obtained. The post-hoc power analysis focused on VV rotation, i.e., the parameter with the lowest absolute difference (10.5°) between BB and squat. With α = 5%, the power was 80.3%.

## Results

### Comparison between knee position at the moment of BB and single leg-squat range of translations/rotations

**Fig. 3:**
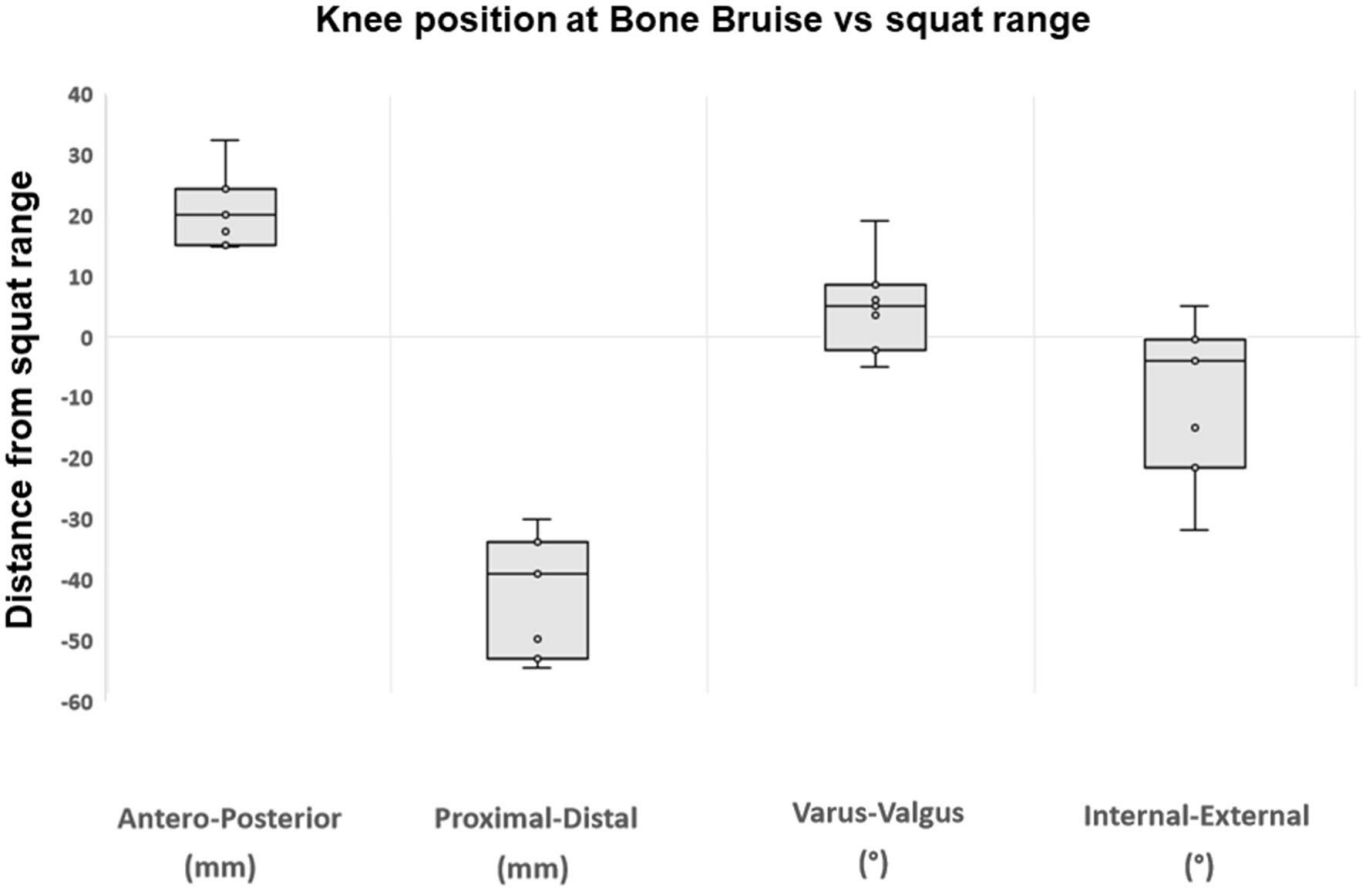
Knee position at the moment of bone bruise compared to the range of motion during single-leg squat. The distance values represent the difference between the upper limit of each patients’ squat range and the knee position/rotation at bone bruise. Positive values represent a more anterior, proximal position, valgus, internal rotation

For all the patients, the knee flexion angle at BB (> 30° in all cases) was within the range of values obtained by dynamic RSA analysis of single-leg squat. All the patients at the moment of BB had an anterior tibial translation exceeding their own maximal translation observed during the single-leg squat (Fig. 3). All the patients showed negative values of PD translation at BB, while a positive value was always reported during the squat tasks. According to the knee reference system, this means that the femur and tibia models overlapped along the proximo-distal axis at BB but not during the squat.

In five out of seven patients, the tibia was more externally rotated at the moment of BB than the maximal external rotation observed during the single-leg squat. In one patient, the tibial rotation at the moment of BB fell within the rotation range during squat, and in one patient, it resulted more internally rotated than the maximal internal rotation of squat. In five out of seven patients, the knee at BB had more valgus than during the entire single-leg squat, while two patients had a more varus alignment compared to the VV range observed during the execution of the squat (Fig. 3).

## Comparison between knee at the moment of BB and single-leg squat position at the same degree of flexion

The statistical comparison between the tibiofemoral position at the moment of BB and the tibiofemoral position during single-leg squat at the same flexion of BB was performed at flexion angles > 30° in all patients (Table 2). The mean AP translation at the moment of BB was significantly higher than during the single-leg squat (more anterior, *p* < 0.001). Furthermore, the mean difference at the moment of BB was significantly different in IE rotation (more external, *p* = 0.04), VV rotation (more valgus, p = 0.03), and proximal–distal translation (*p* < 0.001) (Table 2).

**Table 2:** Comparison between BBs genesis and single-leg squat at the same flexion angle

	BBs genesis	Squat at BBs flexion	Difference [95% CI]	*p *value
AP (mm)	46.9 ± 2.8	18.5 ± 2.5	28.4 [25.3–31.5]	< 0.0001*
PD (mm)	−22.9 ± 1.3	26.5 ± 4.2	49.4 [45.8–53.0]	< 0.0001*
IE (°)	−6.1 ± 3.7	7.6 ± 5.0	13.7 [8.6–18.8]	0.04*
VV (°)	4.1 ± 2.4	−6.4 ± 3.8	10.5 [6.8–14.2]	0.03*

*CI* confidence interval, *AP* antero-posterior translation, *PD* proximo-distal translation, *IE* internal–external rotation, *VV* varus–valgus

*Statistically significant differences (*p* < 0.05)

## Discussion

**Fig. 4:**
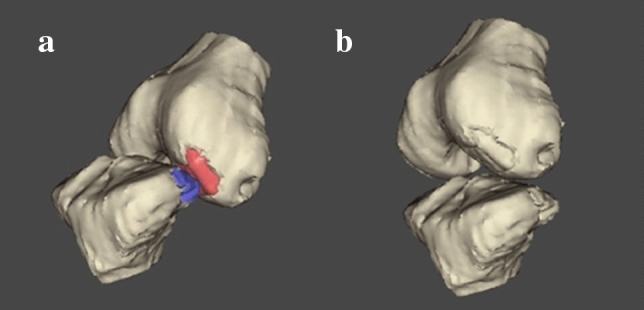
Comparison of knee position at BB genesis (**a**) and during squat for the same flexion angle of BB genesis (**b**). Notice the significant amount of tibial anterior and proximal translation in the first figure with respect to the knee position and orientation observed in the execution of a physiological motor task, reflecting the idea that BB genesis could occur during a knee locked sub-luxation

The most important finding of the present study was that BB in noncontact ACL injuries seems to occur with flexed knee (average 46°), a significant amount of anterior and proximal translation of the tibia, and mainly in external rotation and valgus. In all BB models, the tibia appeared evidently sub-luxated anteriorly. This condition became more evident when compared to Dynamic RSA at the same knee flexion angles (Fig. 4). Moreover, knee kinematics during an active flexion–extension did never reproduce the BB mechanisms.

The present study was one of the firsts that reproduced a 3D matching of the tibiofemoral joint position at the moment of BB and the first one that compared such position to a weightbearing dynamic task performed by the same patients.

Interesting considerations could be outlined based on the findings of the present study regarding the current theories of ACL injury mechanisms. One of the most influential is considered the one proposed by Koga et al., which performed a 3D video-analysis on real in-vivo noncontact ACL injuries [18, 19]. The authors identified 3 phases: (1) axial and knee valgus loads are applied on early-flexed knee resulting in lateral tibial compression; (2) the compressive force coupled with anterior force vector caused by quadriceps contraction causes the lateral femoral condyle to shift posteriorly, and the tibia to translate anteriorly and to rotate internally (ACL rupture); (3) the medial femoral condyle displaces posteriorly, resulting in external rotation of the tibia while the knee flexes [19]. Examining the data provided by the authors [18], the plateau of anterior tibial translation seems to occur approximately between 50 and 100 ms after initial ground contact, with the knee flexed between 40° and 60° and when tibial rotation is reversing from internal to external rotation. This translational and rotational pattern described in the late phase of the injury mechanism seems consistent with the pattern reported at the time of bone bruise described in the present study. Thus, BB could occur after ACL rupture, when the tibial and femoral motion is guided by the abnormal kinematics due to the ligamentous injury and the traumatic inertial energies, exposing to contact articular surface that would not be normally overlapped during physiological motion. Moreover, in the present study, the translation and rotations described in the moment of BB are not included in the normal ranges of translation\rotations found with dynamic RSA when performing squat. Based on these considerations, it could be speculated that BB do not occur within the first 40 ms of ACL rupture when knee is early flexed and internally rotated, but in the following frames, when the ligament is already gone and the tibia is anteriorly and proximally subluxated.

**Fig. 5:**
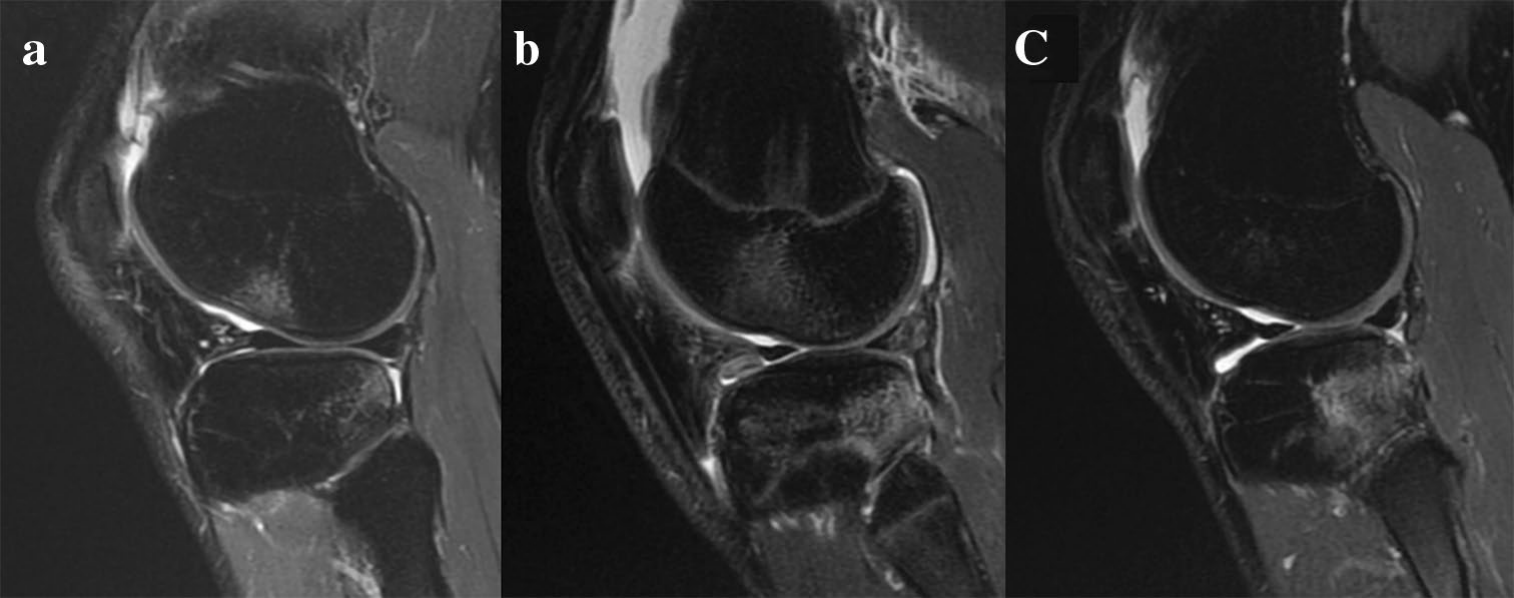
**a–c** MRI images of lateral BB from the case series*.* BB areas are predominantly located in posterior aspects of tibial plateau (inferiorly respect to articular cartilage and subchondral bone) and at the center of lateral femoral condyle

The findings of the present study are in contrast with ones reported in previous studies with similar methods [17, 23]. Kim et al.[17], evaluating 8 patients with bicompartmental BB, reported that 12° of knee flexion, 22 mm of anterior translation, 15° of internal tibial rotation, and 5° of valgus rotation (5°) were present in the BB position relative to rest MRI position. The main methodological difference, which could explain these substantial differences, is the knee starting position used in the cited study to calculate data describing BB knee orientation. The choice of MRI static knee position as reference could cause measurement bias, for the absence of weightbearing and the intra-subject variability of rest position during imaging acquisition [15]. Moreover, according to BB pattern and distribution described in the literature [24, 29], their position seems to be localized predominantly in posteromedial and postero-lateral tibial plateau, even more posteriorly (and inferiorly) with respect to the articular cartilage and subchondral bone (Fig. 5). It could be hypothesized that an impact in such a steep and “vertically-oriented” area would occur with higher knee flexion values than those reported by Kim et al. [17].

The more recent study by Shi et al. [25] represented an evolution of the methodology of the above-mentioned research. With a greater sample size and numerical optimization of BB identification and matching, based on signal intensity on MRI, they claimed bone contusion occurred at an average knee flexion of 36.1°, anterior tibial translation of 34.3 mm, external rotation near 10°, and valgus near 10°. These data are extremely consistent with the ones reported in the present study and highlighted the role of higher values of knee flexion than ones reported by Kim et al. [17, 23]. Nevertheless, the matching of tibia and femur was based only on lateral compartment BB, thus possibly affecting the reliability of tibial internal–external rotation values. Indeed, from a geometrical point of view, it would be impossible to obtain a single tibiofemoral position at BB if only a single compartment is involved, without any hint on how to constrain the contralateral compartment.

The present study has several limitations. First, the sample was relatively small. Thus, the inferences drawn are far from being conclusive. The need for bicompartmental BB in both tibia and femur for optimal bone matching considerably reduced the number of patients available. Indeed, bone edema on medial femoral condyle was reported in approximately 8% of knees with ACL injury [12], which is in accordance with the numbers of the present study (11%). Moreover, the sample size was similar to the only other study that used bicompartmental BB for 3D bone matching (8 patients, [17]). These aspects restrict the findings only to the subset of ACL injuries with these features. However, the predominant knee position at BB and the gross differences from in-vivo kinematics identified in the present study could contribute to enlarging the knowledge on BB mechanism by partially overcoming previous technological limitations.

Another element to clarify, which should be a matter of further research, is that the present study -as the other studies with similar methodologies [17, 22, 23]- is based on the assumption that medial and lateral BB occurs in the same moment. No proofs of this -and the contrary—are currently available, even if some authors imputed the medial bone bruise to a countercoup mechanism that occurs during backward tibial reduction after anterior subluxation [16]. Another consideration on BB should be made and possibly clarified in the future: since the present study was based on exact matching of bone edema areas, it is assumed that those areas effectively represent the exact point of bone-to-bone impact. Whether this is actually true, or if the edema distribution is determined by other factors such as subchondral and cancellous bone architecture or mechanical properties, water content distribution, or biological healing, remains unknown.

## Conclusions

Based on the knee position found in the present study and from the comparison with in-vivo kinematics, bone bruise occurs out of physiological range of motion and could reflect the late phase of noncontact ACL injury rather than the mechanisms leading to ligament failure. These findings suggest that caution should be used when interpreting BB to understand ACL injury dynamics.

## Abbreviations

ACL: Anterior cruciate ligament
AP: Anterior–posterior
BB: Bone bruise
IE: Internal–external
MRI: Magnetic resonance image
PD: Proximal–distal
RSA: Roentgen stereophotogrammetric analysis
VV: Varus–valgus
